# Possible Roles of the Dominant Uncinate Fasciculus in Naming Objects: A Case Report of Intraoperative Electrical Stimulation on a Patient with a Brain Tumour

**DOI:** 10.3233/BEN-110249

**Published:** 2013

**Authors:** Keiko Nomura, Hiroaki Kazui, Hiromasa Tokunaga, Masayuki Hirata, Tetsu Goto, Yuko Goto, Naoya Hashimoto, Toshiki Yoshimine, Masatoshi Takeda

**Affiliations:** ^1^Department of PsychiatryOsaka University Graduate School of MedicineSuita-cityOsakaJapan; ^2^Department of NeurosurgeryOsaka University Graduate School of MedicineSuita-cityOsakaJapan

**Keywords:** Left uncinate fasciculus, naming objects, awake surgery, intraoperative electrical stimulation, low-grade astrocytoma

## Abstract

How the dominant uncinate fasciculus (UF) contributes to naming performance is uncertain. In this case report, a patient with an astrocytoma near the dominant UF was given a picture-naming task during intraoperative electrical stimulation in order to resect as much tumourous tissues as possible without impairing the dominant UF function. Here we report that the stimulations with the picture-naming task also provided some insights into how the dominant UF contributes to naming performance. The stimulation induced naming difficulty, verbal paraphasia, and recurrent and continuous perseveration. Moreover, just after producing the incorrect responses, the patient displayed continuous perseveration even though the stimulation had ended. The left UF connects to the inferior frontal lobe, which is necessary for word production, so that the naming difficulty appears to be the result of disrupted word production caused by electrical stimulation of the dominant UF. The verbal paraphasia appears to be due to the failure to select the correct word from semantic memory and the failure to suppress the incorrect word. The left UF is associated with working memory, which plays an important role in recurrent perseveration. The continuous perseveration appears to be due to disturbances in word production and a failure to inhibit an appropriate response. These findings in this case suggest that the dominant UF has multiple roles in the naming of objects.

## 1. Introduction

The uncinate fasciculus (UF) is a white matter tract that connects the inferior frontal lobe with the anterior inferior temporal lobe [1]. A tumour resection study revealed that the left UF is essential for naming common objects [2]. Also, a diffusion tensor imaging (DTI) study found that demyelination and axonal injury of the left UF were associated with a decline in naming performance [3]. Although these two studies suggested that the left UF is associated with naming performance, they have a few shortcomings. In the tumour resection study, not only the left UF but also a part of the surrounding cortical regions was resected [2]. In the previous DTI study, the patients had temporal lobe epilepsy [3], which is likely to cause atypical language lateralization [4]. In addition, the DTI study did not assess whether the left UF was associated with symptoms that are related to naming deficits, such as paraphasia, perseverations, and speech arrest [3].

ISSN 0953-4180/13/$27.50 © 2013 – IOS Press and the authors. All rights reserved

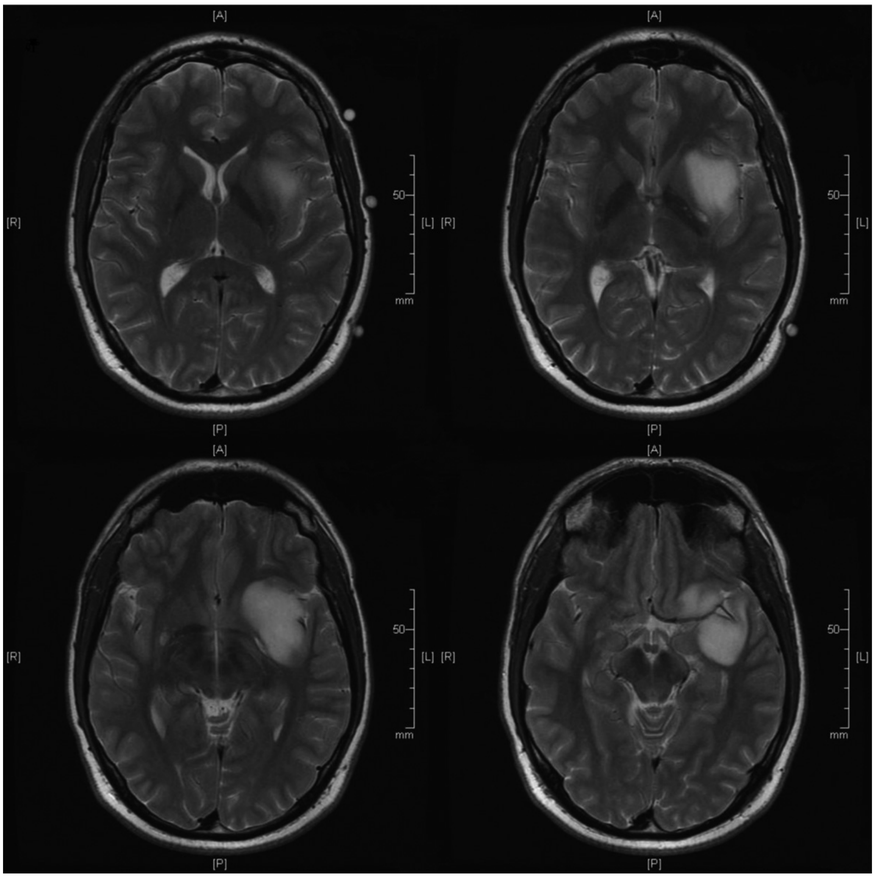

Fig. 1. Preoperative transaxial T1-weighted MR images showing a left frontotemporal low-grade astrocytoma, which involved the insula, temporal stem, and orbitofrontal cortex. A: anterior, P: posterior, R: right, L: left.

Intraoperative electrical stimulation inhibits the function of a restricted region of the brain [5], which makes it possible to observe real-time responses when the region has been functionally inhibited by the stimulation. There have been three reports on the effects of intraoperative stimulation in the dominant UF, each reporting a different reaction: semantic paraphasia [6], phonetic paraphasia [2], and no language disturbance [7]. Although two of the studies [2,6] showed that the dominant UF is involved in naming performance, the types of naming errors differed between the two studies. Therefore, how the dominant UF is involved in naming performance is still uncertain. In the present study, intraoperative stimulation was used to assist in the resection of a tumour near the dominant UF. Here, we report that the patient showed multiple different symptoms related to naming objects during intraoperative stimulation of the dominant UF that provide some insights into how the dominant UF is involved in naming objects.

## 2. Methods

### 2.1. Patient

The patient was a 39-year-old Canadian man who had come to Japan in 2003 to teach English. He graduated from a college in Canada and spoke English as a first language. Following a motorbike accident, a routine computed tomography scan detected a tumour on the left insula, temporal stem, and orbitofrontal cortex. However, brain magnetic resonance (MR) images did not show contrast enhancement, so that the tumour was suspected to be benign and was followed up annually. Three years later, the tumour seemed to be larger, and was suspected to be a low-grade astrocytoma (Fig. 1). All preoperative and postoperative neuropsychological tests and an intraoperative naming test were performed in English. He was 100 percent right-handed as measured by the Edinburgh Handedness Inventory. According to the Magnetoencephalography and thiamylal

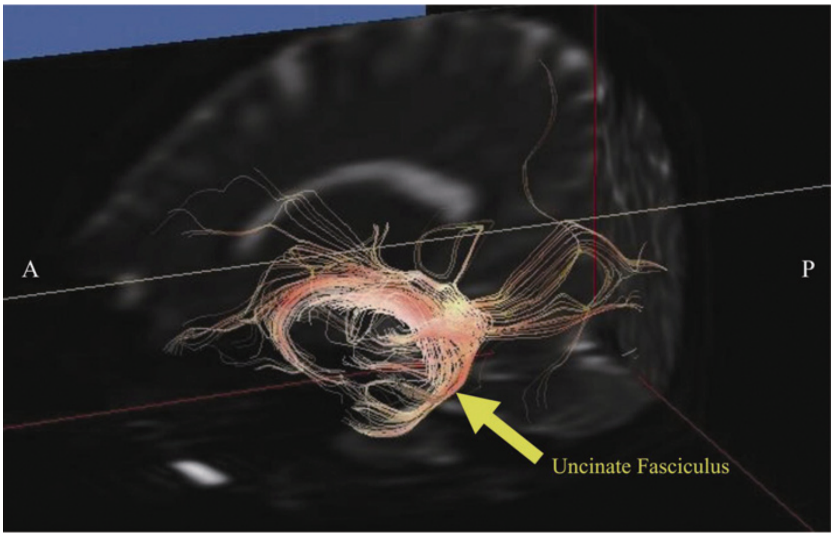

Fig. 2. Preoperative fibre tracking used to set the region of interest in the left temporal stem. The tract that is hooked at the left anterior temporal region was the left uncinate fasciculus (yellow arrow). The tract running backward is the left optic radiation. A: anterior, P: posterior.

sodium Wada Test with language tasks, his language functions were lateralised to the left hemisphere. The Wada Test also revealed that both hemispheres were involved in verbal memory. He experienced no seizure episodes or behavioural changes prior to the tumour resection. Written informed consent was obtained from the patient for publication of this report.

### 2.2. *Preoperative cognitive performance*

Preoperatively, the patient complained about mild word-finding difficulties only when he was talking with native English speakers on business, but his colleagues did not make any remarks about it. He scored 30/30 on the Mini-Mental State Examination (MMSE). In the Western Aphasia Battery (WAB), he scored an aphasia quotient (AQ) of 99.6, a language quotient (LQ) of 99.8, and a cortical quotient (CQ) of 99.0, respectively. These results of the examinations indicated that his cognitive performance was not impaired.

### 2.3. *Preoperative magnetic resonance-diffusion tensor imaging data acquisition and processing*

Preoperatively, anatomical MR images and DTIs were acquired on a 3.0 Tesla MR whole-body imager (Signa VH/i, GE medical Systems, Milwaukee, WI, USA). Three-dimensional fibre tracking (FT) based on the DTI data was performed using Volume-One and dTV software (free software by Masutani, URL: http://www.ut-radiology.umin.jp/people/masutani/dTV.htm). The UF tractography was performed using a two-region of interest (ROI) method in the same way of a previous study [8]. The seed ROIs were placed in the anterior part of the UF in the coronal plane at the level of the anterior portion of the genu of the corpus callosum that was anterior to the anterior horn of the lateral ventricle. The target ROIs were placed in the white matter in the coronal plane at the most anterior part of the temporal stem. The colour-coded maps were employed to precisely and objectively place these ROIs into the UF tracts. To determine reconstructed coronal sections at the level of the genu of the corpus callosum, a reconstructed sagittal section of the colour-coded map was employed. Figure 2 shows the left UF with the FT images.

### 2.4. *Intraoperative electrical stimulation*

A Stryker Navigation System (Stryker, Kalamazoo, MI, USA) was used with 1.4 mm thin-slice sagittally sectioned MR images, which the FT had been superimposed on, for the navigation. After the main mass of the tumour was resected, electrical stimulation in combination with a picture-naming task was performed under local anaesthesia to determine whether additional tumorous tissue could be resected without impairing its function. The stimulation point abutted the posterior limb of the internal capsule and was very close to the left UF (Fig. 3). An Ojemann bipolar stimulator with 5 mm spaced tips was applied to deliver a biphasic current. The electrical stimulation was 6 mA. Each

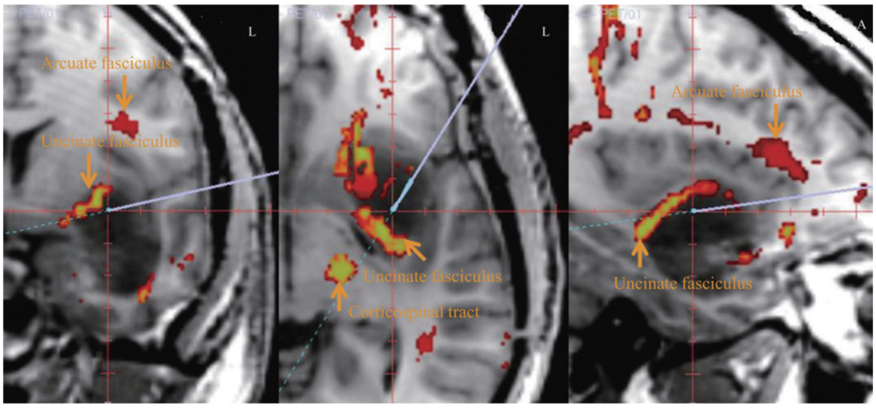

Fig. 3. Intraoperative neuronavigation pictures, in which the preoperative two-dimensional fibre tracking was projected on transaxial T1-weighted MR images (Left: a coronal image, Middle: a transaxial image, and Right: A sagittal image). The blue lines point to where the electrical stimulation with a picture-naming task was provided. L: left, A: anterior.

stimulation consisted of biphasic square wave pulses of 0.2 millisecond single phase duration at 50 Hz with the maximal train duration of five seconds. As Fig. 3 shows, we intermittently stimulated the same point at a distance of within 5 mm from the dominant UF. An electrical stimulation of 6 mA was shown to reach 5 mm from the stimulation point [9]. The electrocorticography activity was monitored to observe spontaneous or after-discharge spikes to minimise the risks of evoking a seizure by continued stimulation and to avoid the possibility of errors caused by the propagated effects of the current [2].

### 2.5. *Picture-naming task*

In the picture-naming task, the patient was asked to name a colour picture of an object that was on the computer screen beside his face. We prepared eight different colour pictures, which the patient could correctly name on a preoperative examination. The picture stimuli were presented repeatedly one after another in the following order: a clock, tire, banana, strawberry, pencil, umbrella, bicycle, and elephant. The picture-naming task was continuously performed while the dominant UF was being either stimulated or not stimulated. The patient was not informed when the dominant UF was being stimulated. The whole picture-naming task took approximately 10 minutes.

## 3. Results

### 3.1. *Intraoperative electrical stimulation*

We performed the picture-naming task eight times under the condition of stimulating the dominant UF. Among the eight times, the patient showed five incorrect responses. The patient stammered twice when he tried to name a picture of an umbrella and a banana respectively. The responses were considered as naming difficulty. When seeing a picture of a strawberry, the patient answered “fish”, which was not in the picture stimuli. A fish and strawberry are not semantically interconnected, and the response was considered as verbal paraphasia. When the patient was shown a picture of an elephant, he answered “umbrella”, which was shown two picture stimuli earlier. The response was considered as recurrent perseveration, which is an unintentional repetition of a preceding response when a new response following an interruption is expected to occur [10]. When asked a second time, he answered “umbrella” again. The response was considered as continuous perseveration, which is an inappropriate repetition of a preceding response without interruption [10]. Moreover, after producing the incorrect responses, the patient always gave the preceding incorrect response even though the stimulation had ended. The responses were considered as continuous perseveration. Except for the continuous perseveration, the patient did not produce any incorrect responses when not being stimulated.

### 3.2. *Postoperative course*

Since the stimulation of the dominant UF produced some naming and related disturbances, the neurosurgeons did not resect additional tissue of the dominant UF. The tumour resection was successful, so that neither chemotherapy nor radiotherapy was postoperatively given. One week after the operation, the patient scored 25/30 on the MMSE with scoring 2/5 on the serial 7's and scoring 1/3 on the recall. Regarding to his language performance, he scored the AQ of 100, the LQ of 100, and the CQ of 99.5, respectively. The patient did not show any naming difficulty, paraphasia, and perseveration. At six weeks after the operation, his MMSE score was completely back to 30/30, and he obtained a verbal memory index of 100, a visual memory index of 126, a general memory index of 106, an attention/concentration index of 102, and a delayed memory index of 105 in the Wechsler Memory Scale-Revised. Hence, his cognitive performances were not impaired by the tumour resection.

## 4. Discussion

When stimulating the dominant UF with the neuronavigation system during the picture-naming task, the patient displayed naming difficulty, verbal paraphasia, and recurrent and continuous perseveration. After producing the incorrect responses and after the stimulation had ended, the patient displayed continuous perseveration.

The findings of this report, in which the dominant UF stimulation caused some deteriorations of naming performance, are consistent with the results of previous tumour resection and DTI studies [2,3]. The left UF connects to the inferior frontal lobe, which is essential for word production [11]. Dysfunction of the dominant UF appears to be the cause of the naming difficulty.

The verbal paraphasia that we observed during the dominant UF stimulation is closely related to previous studies that found phonetic paraphasia and semantic paraphasia during the dominant UF stimulation with a picture-naming task [2,6]. The dominant anterior inferior temporal lobe, to which the UF connects [1], is involved in semantic memory that is information about the concept or meaning of words and objects [12,13]. Moreover, the UF is involved in verbal planning and suppression [14]. Hence, verbal perseveration should be caused by disturbances in selection of a semantically correct word and failures to suppress the inappropriate word.

Recurrent perseveration and continuous perseveration, which were induced by the dominant UF stimulation during the picture-naming task in this case, have not previously been reported to be caused by the dominant UF stimulation. Recurrent perseveration is initiated with an unsuccessful search in semantic memory for the correct word, and then a recently heard word can be selected from short-term memory [15]. A word, which has just been articulated, should be temporarily held in working memory (WM), with which the UF is associated [16]. In this case, the stimulation must have prevented the patient from retrieving a word from semantic memory, so that the patient incorrectly must have selected another word that had been recently articulated from WM.

Continuous perseveration was observed when motor output was disturbed [10] and was associated with a failure to inhibit an appropriate response [17]. Therefore, electrical stimulation of the dominant UF, which is involved in word production [11] and motor suppression [14], could produce continuous perseveration. Although it is unclear why continuous perseveration occurred just after switching off the stimulation, it was always observed after the patient produced the incorrect responses with the stimulation. The stimulation, which was enough to produce incorrect responses, might have produced continuous perseveration.

Several issues should be considered when generalising the findings of this case report. First, we need to acknowledge that we provided the picture-naming task only eight times under the condition of stimulating the UF, so that we were unable to examine whether the same incorrect responses can be observed repeatedly. Second, the tumour seemed to shift the UF medially, so that the UF may have not been placed in the expected anatomical region. Third, whenever the intraoperative neuronavigation system is employed, the possibility of an intraoperative brain shift should be considered [18, 19] because the brain shift may result in inaccuracy of stereotactic image guidance on the preoperatively acquired brain images [18] and may reduce reliability of the neuronavigation system [19]. However, the displacements of deep tumour margins or subcortical structures are not as pronounced as those of superficial or cortical structures [19,20]. Furthermore, experienced neurosurgeons identified the tumour and differentiated the brain structures including the cortices, white matter tracts, and deep brain structures based on their anatomical knowledge. Finally, we may have failed to depict the inferior occipitofrontal fasciculus (IOFF) independently from the UF because it is very difficult to distinguish these two regions in FT images [21]. Because the UF tractography method that we used has been shown to be reliable [8], we believe that we properly depicted the UF. In addition, because our stimulation point was very close to the UF (Fig. 3) and because the electric current we applied was enough to reach 5 mm from the stimulation point, the electrical stimulation must have reached the UF. Hence, we are confident that we properly stimulated the dominant UF instead of the IOFF.

In this case, stimulating the dominant UF caused several responses (naming difficulty, verbal paraphasia, and recurrent and continuous perseveration). These findings suggest that the dominant UF is related to multiple roles in the naming of objects.
